# Implementation of an IMU Aided Image Stacking Algorithm in a Digital Camera for Unmanned Aerial Vehicles

**DOI:** 10.3390/s17071646

**Published:** 2017-07-18

**Authors:** Ahmad Audi, Marc Pierrot-Deseilligny, Christophe Meynard, Christian Thom

**Affiliations:** 1 Université Paris-Est, IGN, LaSTIG, LOEMI, 73 Avenue de Paris, 94160 Saint-Mandé, France; Christophe.Meynard@ign.fr (C.M.); Christian.Thom@ign.fr (C.T.); 2 Université Paris-Est, IGN, LaSTIG, LOEMI, ENSG, 6-8 Avenue Blaise Pascal, 77420 Champs-sur-Marne, France; Marc.Pierrot-Deseilligny@ensg.eu

**Keywords:** UAVs, stacking, inertial measurement unit, image processing, photogrammetry, real-time, co-design

## Abstract

Images acquired with a long exposure time using a camera embedded on UAVs (Unmanned Aerial Vehicles) exhibit motion blur due to the erratic movements of the UAV. The aim of the present work is to be able to acquire several images with a short exposure time and use an image processing algorithm to produce a stacked image with an equivalent long exposure time. Our method is based on the feature point image registration technique. The algorithm is implemented on the light-weight IGN (Institut national de l’information géographique) camera, which has an IMU (Inertial Measurement Unit) sensor and an SoC (System on Chip)/FPGA (Field-Programmable Gate Array). To obtain the correct parameters for the resampling of the images, the proposed method accurately estimates the geometrical transformation between the first and the *N*-th images. Feature points are detected in the first image using the FAST (Features from Accelerated Segment Test) detector, then homologous points on other images are obtained by template matching using an initial position benefiting greatly from the presence of the IMU sensor. The SoC/FPGA in the camera is used to speed up some parts of the algorithm in order to achieve real-time performance as our ultimate objective is to exclusively write the resulting image to save bandwidth on the storage device. The paper includes a detailed description of the implemented algorithm, resource usage summary, resulting processing time, resulting images and block diagrams of the described architecture. The resulting stacked image obtained for real surveys does not seem visually impaired. An interesting by-product of this algorithm is the 3D rotation estimated by a photogrammetric method between poses, which can be used to recalibrate in real time the gyrometers of the IMU. Timing results demonstrate that the image resampling part of this algorithm is the most demanding processing task and should also be accelerated in the FPGA in future work.

## 1. Introduction

Over the last decade, Unmanned Aerial Vehicles (UAVs) have been widely used for civil applications [1], especially for environmental surveys [2,3]. Their growing popularity has led the LOEMI (Laboratoire d’Opto-électronique, de Métrologie et d’Instrumentation) team of Institut national de l’information géographique (IGN)/LaSTIG (Laboratoire des Sciences et Technologies de l’Information Géographique) to design and produce an ultra-light smart digital aerial camera, more suitable for photogrammetric applications than consumer cameras.

This camera was originally used to exploit photogrammetric and metrological surveys using UAVs while some research works were already underway, such as [4,5]. The next targeted applications involve shots under cloudy conditions, night-time surveys and narrow spectral bandwidth imagery, which usually imply a long exposure time. Images acquired with a long exposure time using a camera embedded on UAVs may exhibit motion blur due to the erratic movements of the UAV. This paper presents an image stacking algorithm that can provide an accurate blur-free composite image of photogrammetric quality with an equivalent long exposure time. An initial presentation of this work has been done in [6].

In this context, the main part of our algorithm is based on feature points image registration [7,8,9]. This technique consists of feature detection, feature matching, transformation estimation, image transformation and resampling. The ultimate objective of our work is to achieve real-time processing of the algorithm, leading to only the resulting image needing to be saved and therefore ensuring a better overall frame rate. The inertial measurement unit mounted inside the camera can gives the relative orientations of the camera during shoots, quantifying an initial amount of motion between poses. This consequently speeds up the expensive computing of matching feature points considerably, which is one of the most time-consuming tasks, making our stacking algorithm more appropriate for real-time performance. Images are acquired with a high frame rate, which implies minor deformation between images.

Two approaches are introduced in this paper. For the first approach, the UAV is assumed to be quasi-stationary during exposures; only camera attitude variations are considered; and only image deformation due to camera rotation is corrected. One interesting by-product of this method is a better estimation of variation in the orientation of the camera between shots, which can be used to recalibrate the IMU gyrometers. Other sensors, such as LiDAR sensors on the UAV, can then benefit from the resulting better quality of the IMU data. The second approach uses the 2D planar homography transformation supposing that not only the 3D rotation is taken into account on camera movement, but also the translation when the scene is nearly planar.

Our algorithm works completely automatically, whether offline or online. Some tests have been completed on several types of UAVs, including the Copter 1B UAV equipped with the IGN camera to assess the accuracy of the method, and some of the results are presented.

## 2. Related Works

This work relates to two fields: image stacking and visual-inertial fusion techniques.

The stacking technique creates a single image by combining many images that may be superimposed. One stacking method is HDR (High Dynamic Range) imaging, which combines multiple images with different exposure levels in order to obtain a single image with a large dynamic range [10,11,12]. These works assume that the images are superimposable; differing from our method on this basis.

Many visual-inertial fusion techniques have been proposed in the literature. We hereby present some of them. The authors of [13] suggests using the inertial measurement sensors to measure the camera’s acceleration and angular velocity during exposure, which can then be used to estimate the blur function and then deconvolve the blurred image to achieve sharp results. Their method only works for very small movements, and many cited limitations, such as sensor accuracy and noise, constrain their usage. In the same context, the IMU is used in [14] during aerial shooting by a camera embedded on an aircraft to eliminate blur and provide a deblurred image. The authors’ objective is close to ours, but their method only covers very small movements. Their results show that images with more than seven pixels in motion blur record minor improvements. Other works relate to the same objective using deblurring algorithms without IMU data: [15,16,17].

Some studies in literature expand on the combination of IMU and computer vision for tracking purposes. For example, a vision-assisted algorithm is combined to improve positioning and to track the performance of mobile devices for SLAM (Simultaneous Localization And Mapping) applications [18]. Parts of this approach seem similar to ours. It uses IMU gyrometer measurements to estimate rotation angles to improve the accuracy of trajectory estimation, but they also use reference nodes as control points identified in the vision processing phase to correct the propagation of IMU errors. Therefore, this method is not optimal if no reference nodes are available along the trajectory. The authors of [19,20,21] use a combination of inertial sensors and a camera for motion tracking in augmented reality applications. Most fusion sensor works use inertial sensors to aid computer vision methods, making the system more accurate and more robust.

Some parts of our work have also been explored in alternative domains. For example, a real-time video stabilization algorithm for unmanned aerial vehicles is proposed in [22], but this method excludes any aided sensors, such as the IMU, to improve the accuracy of motion estimation or to reduce the tracking time for features in consecutive frames.

## 3. Materials and Methods

### 3.1. Hardware

**Figure 1:**
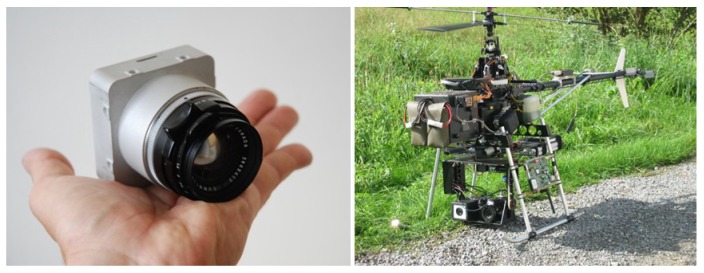
Institut national de l’information géographique (IGN) camera with the Copter 1B UAV developed by Wikhydro.

**Figure 2:**
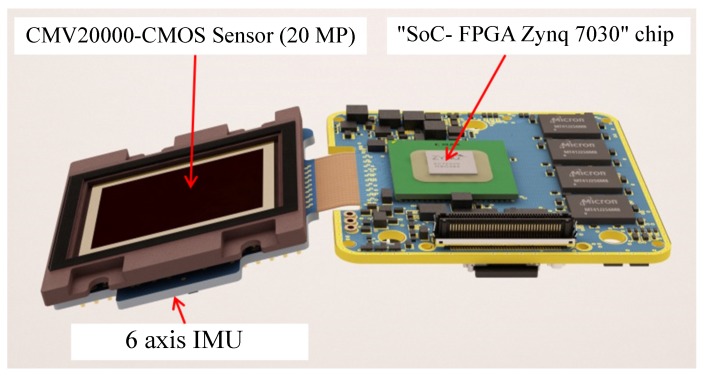
The CMOS sensor with the inertial sensors and the embedded intelligence.

The system presented in Figure 1 consists of a UAV equipped with an IGN lightweight photogrammetric camera [23]. The camera used to implement our algorithm is shown in Figure 2.

This camera was developed at the French National Mapping Agency (IGN) by the LOEMI team. It is based on a global shutter CMOS 20MP sensor (CMOSIS CMV20000) from CMOSIS, Anvers, Belgium, and a Zynq 7030 SoC/FPGA from Xilinx, San Jose, CA, USA, allowing some image processing algorithms to be implemented in hardware, making them real-time compliant. It has both embedded intelligence, in the form of two ARM cortex A9 CPUs running a Linux OS and application software, and a programmable logic subsystem originally used to acquire image data from the sensor. A standard 35-mm optical lens is used for this purpose. The camera prototype we used was fitted with a Bayer sensor and has a resolution of 20 MP (5120 × 3840). Our work aims to be used for a hyperspectral imaging system consisting of several monochrome IGN cameras; thus, we use the 5-MP (2560 × 1920) grayscale images obtained from the original pixel-stream by averaging each green pair of the Bayer pattern.Recently, LOEMI team engineers integrated a GNSS (Global Navigation Satellite System) receiver, which can be used in future works to take into account UAV translation during shots.

**Figure 3:**
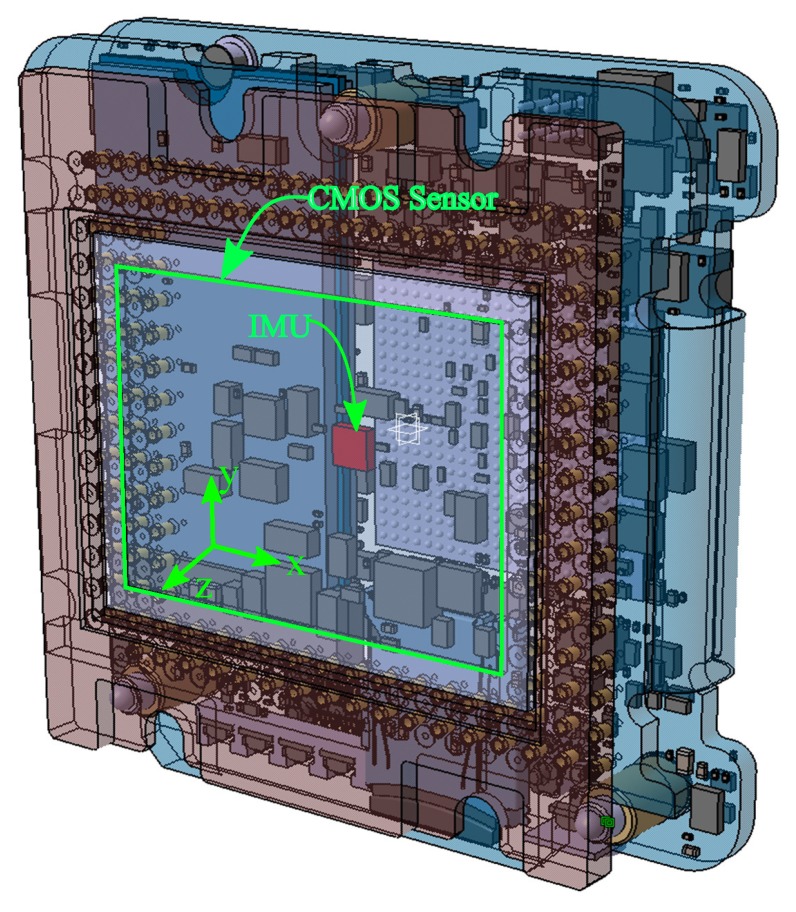
The IMU and the CMOS sensor inside the IGN camera.

The ICM-20608-G IMU sensor from InvenSense used in the camera is a low-cost MEMS (Micro-Electro-Mechanical System) device. It measures angular speed around the three axes with low latency and at a high frequency. The main disadvantage of MEMS technology is the reduced performance in terms of accuracy and stability: the slow evolving bias is the main type of error that affects measurements, as it accumulates over time due to the integration of angular velocity. We will show that it can be estimated by sensor fusion and then eliminated from the subsystem. Laboratory thermal calibration is used to eliminate the temperature effects caused by the dissipation of heat inside the camera (75 °C). The IMU sensor is tightly fixed inside the camera to ensure that both sensors experience the same 3D rotation as shown in Figure 3. We use the IMU subsystem to obtain the estimation of the absolute 3D rotation of each pose ($R_{imu}^{n}$) acquired by the CMOS sensor.

### 3.2. Calibration of the Camera

In photogrammetric processes, cameras should be calibrated to take accurate 3D geometric measurements from their images. This calibration involves estimating their internal orientation. This is obtained using the camera self-calibration bundle adjustment method via MicMac, a free open-source photogrammetric software developed by IGN [24]. The method used in MicMac is based on SIFT points and the matching relationship in stereo images. It is applied to each pair of multiple view images taken using an un-calibrated camera. We use the radial standard distortion model [25] in our work.

### 3.3. Images and Data Processing

This algorithm is implemented and executed in the camera and is intended to benefit from the FPGA acceleration. It was initially developed on a desktop PC.

#### 3.3.1. Algorithm

**Figure 4:**
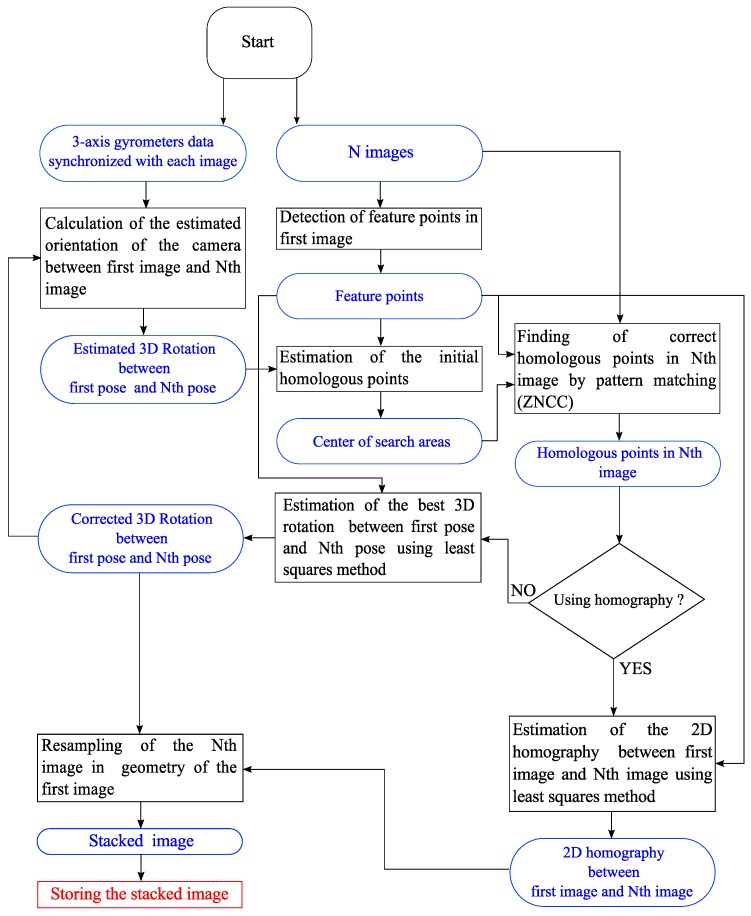
Architecture of the algorithm.

The architecture of the algorithm is illustrated in Figure 4.

- Camera attitude is computed using measurements from the IMU subsystem.
- Acquisition of a burst of 10 images by the camera, saved in RAM at the maximum frame rate of the CMOS (30 images/s); the time difference between successive images is 33 ms.
- Detection of a reasonable number of feature points in the first image. This part is implemented in hardware.
- For the next sequential images:
  In this image, the predicted positions of feature points are computed using IMU orientation to speed up the next step.
  Template matching is used to determine the accurate homologous position of these feature points.
    (a) 3D rotation between the two poses is estimated using the least squares method with outliers elimination. This information may possibly be used as feedback in the IMU subsystem to improve its overall quality by the real-time determination of sensor drifts.
    (b) 2D homography transformation between the two images is estimated using the least squares method with outliers’ elimination.
  The image is resampled in the geometry of the first image.
  Stack it.
- Save the stacked image.

#### 3.3.2. Features Detection

The detection of features is a fundamental task in computer vision applications, such as motion detection, object recognition and tracking, 3D reconstruction, visual odometry, etc. A feature can be defined as a pattern or distinct structure with special properties found in an image. Most of the time, these features are used in further processing steps. In our work, the feature detection algorithm was mainly selected on the basis of its ability to find a reasonable number of good features in a single image (leading by correlation to unambiguous matches in other images) and by the possibility for easy implementation in hardware. After analyzing the main existing feature detectors [26,27,28], we selected the FAST detector algorithm developed by [29].

This detector is many times faster in software than other existing detectors [30], proving that it is conceptually simple, leading to the fact that it can be easily implemented in hardware. In our application, we will not consider the stability of the detector because feature points will only be detected in the first image and then searched for by template matching in other images. Another advantage is that FAST requires far less resources than other detectors and, in parallel, offers sufficient performance for our application.

On the other hand, the FAST detector is dependent, as many other detectors, on a threshold, which must be set according to the image noise level, especially as FAST has been shown to be sensitive to high-level noise [26,27]. However, in our camera, the noise level is reduced to be constant in RMS with respect to gray levels by using the non-linear LUT (Look-Up Tables), which translate the 12 bits of pixel data into 8 bits. We can then use a threshold value independent of image brightness level and content.

#### 3.3.3. Features Selection Approach

The authors of [31] show that FAST tends to detect a high number of feature points in high textured zones, but not in low textured zones. This means that the feature points may be concentrated only in high textured zones or in a specific zone of the image. This non-uniform distribution of points makes the estimation of transformation (rotation or homography) between images less accurate [32]. To limit the high number of feature points in high textured zones and ensure, if possible, that even low textured structure zones may obtain feature points when using a low threshold value, we use a grid-adapted feature selection approach that can maintain a compromise between achieving a more uniform distribution of feature points over the image and implementing this approach in hardware in a simple way.

**Figure 5:**
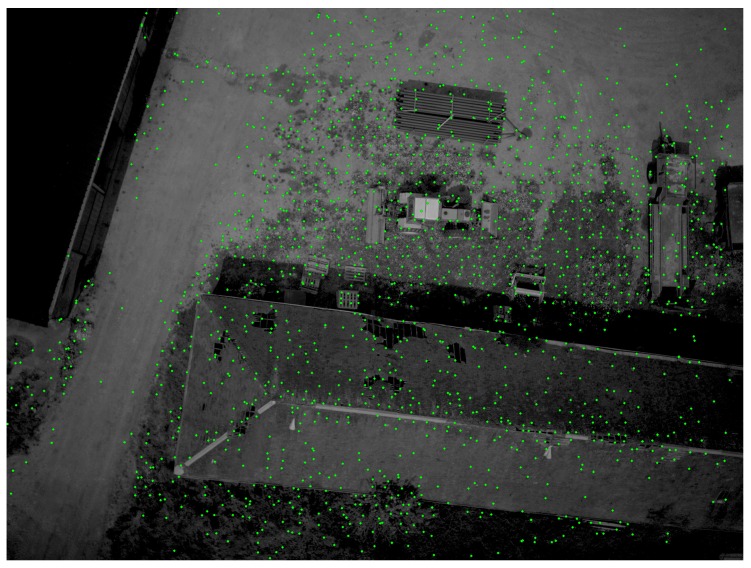
Uniform distribution of features over the image. The threshold intensity value of the detector is set to 7. This image is acquired with a low exposure time, but brightened artificially to clearly show the feature points. The size of the grid-adapted features is set to 100 × 100 blocks.

This approach involves partitioning the image into blocks and only retaining the first feature found by the detector in each block. It is easy to implement in hardware and can improve the uniformity of the distribution of the features over the image as shown in Figure 5: we can see that no feature points are detected in very low textured zones, but a grid-like pattern is observed in high textured zones where a high number of potential feature points are shown.

#### 3.3.4. The FAST Algorithm

**Figure 6:**
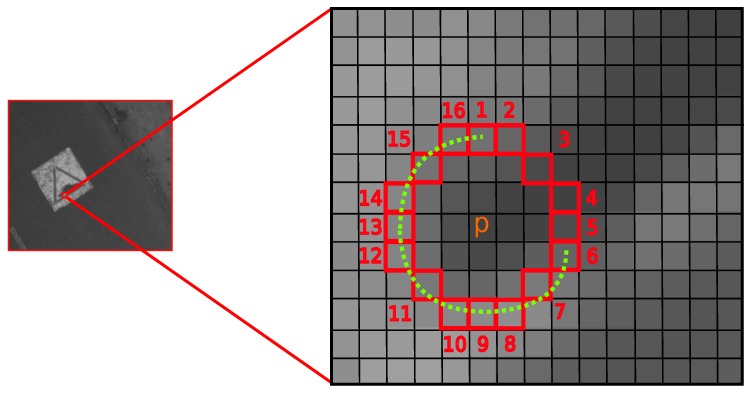
Features from Accelerated Segment Test (FAST)-12 image feature detector illustration.

Features from the FAST detector are based on a 7 × 7 pixels neighborhood centered on the candidate pixel *p*. The size (7 × 7) of the window is chosen according to the original implementation of the FAST detector. The original FAST-*N* algorithm compares the intensity of a feature candidate *p* with each pixel on a 16-pixel Bresenham circle surrounding *p*. The candidate pixel is detected as a feature point if *N* contiguous pixels on the Bresenham circle with the radii *r* all brighter or all darker than *p* by a threshold *t*. Originally, *N* was chosen to be 12 as shown in Figure 6.

##### Hardware Implementation

Processing high resolution images is considerably expensive in terms of computing. The SoC/FPGA integrated into the IGN lightweight photogrammetric camera can provide a suitable solution to speed up this processing. Thanks to this SoC that combines dual ARM cores and an FPGA, hardware/software can be co-designed allowing some processing-intensive tasks to be executed using the programmable logic array and leaving the CPUs free to be used for other tasks. Several hardware implementations have been proposed for FAST. For example, the authors of [33] not only test if candidate pixels are considered as feature points, but also compute a corner score for every feature detected for use in another module called NMS (Non-Maximal Suppression) to reduce the occurrence of adjacent features. Here, we present a simpler hardware implementation of FAST aiming to detect good feature points for the next processing stages with the addition of the selection of grid-adapted features.

##### Hardware Architecture

The design has the following characteristics:

- Image dimensions and threshold configured by software.
- Input pixels format: 8-bit grayscale value.
- Input rates are constant at 1 pixel/clock. For now, the image is read from the memory for debugging reasons. We could improve the design to process the stream of pixels directly from the sensor to achieve real-time performance.
- The output (x,y) coordinates of the feature points (2 × 16 bits) are written to memory.

The design is divided into two modules: the FAST detector and the selector. The FAST detector module computes a validity flag for each pixel under investigation. The selector module picks the first valid feature detected by the FAST detector module in each block of the grid-adapted image.

The “FAST detector” module is divided into two modules: the “(7 × 7) window” and the “corner test”.

We need simultaneous access to the 16 pixels located on the Bresenham circle and the candidate centered pixel, requiring a 7 × 7 window containing the 17 pixels under investigation. For this purpose, we used 6 arrays as FIFO buffer whose depth is equal to the width of the image. Furthermore, we used (7 × 7) 8-bit registers to preserve the intensity values of the window pixels to be processed by the “corner test” module.

The “corner test” module only computes the 12 contiguous pixels with an intensity value greater than the candidate pixel intensity value plus the threshold, excluding those with an intensity value less than the candidate pixel intensity value minus the threshold. This is sufficient to obtain a reasonable number of good feature points. In the first clock cycle, the intensity value of each pixel located on the Bresenham circle is compared with the intensity value of the centered candidate pixel plus the threshold. The output of these 16 comparisons forms a 16-bit wide logic sequence of ‘0’ and ‘1’: ‘1’ if the intensity value of the pixel in question is greater than the constant and ‘0’ in all other cases. In the next clock cycle, the 16-bit sequence is simultaneously compared with the 16 rotations of 12 contiguous ‘1’ and 4 contiguous ‘0’. The output (validation signal) of the “corner test” module is ‘1’ if this sequence matches one of the 16 combinations and ‘0’ in all other cases. Therefore, the output coordinates are delayed by two-clocks.

The “selector” module uses a flag register to deactivate the other valid features in each block. At the beginning of each grid row, the flag register is initialized to *‘0’*. This module also controls the end of image by testing row and column input data. At the end of the image (last block), it sets the column and row output data to *‘0’* and the feature valid output to *‘1’*, so we can detect the end of the features list.

##### The Global Design

**Figure 7:**
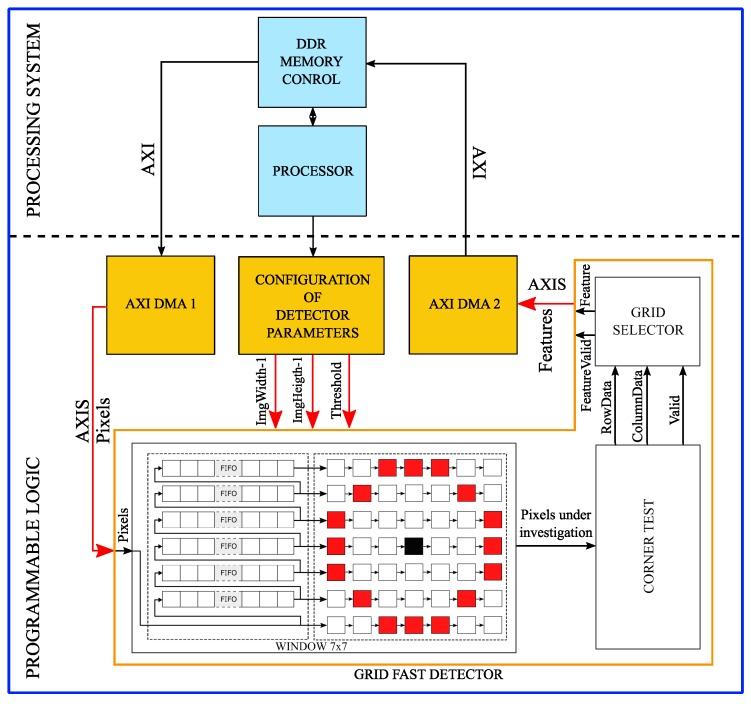
The global design of the grid-based fast detector implementation.

The global design presented in Figure 7 was implemented and tested on the camera Xilinx/Zynq. Our solution uses the standard DDR RAM memory for storing both the image and coordinates of feature points. The AXI bus was used to connect the processor system and memory: the AXI bus provides direct access to the DDR memory. The AXIS transmits stream data between modules in master/slave modes. As a first step, to verify the design of this algorithm, the image in the RAM is read by the first DMA engine and transmitted to the “grid-based FAST detector” module. The resulting feature points coordinates are written back in the memory by the second DMA engine. The timing result of the global design execution is 0.024 s as shown in Table 2; this is the time taken to transfer 2560 × 1920 pixels from the RAM. In the final design, the process will be applied directly to the stream of pixels from the sensor.

##### Resources Usage for the FAST Detector Design

Table 1 illustrates the number of resources used in the FPGA for the implemented design. Compared with other proposed hardware implementations, our design uses far fewer FPGA resources. As an example, the architecture presented in [33] uses about 10-times more look-up tables because they not only test if the candidate pixel is a feature point, but also calculate a corner quality score to use it in another module in order to select the feature point with the maximum score from other adjacent feature points.

### 3.4. Estimation of the Predicted Positions of Homologous Points in Images

**Figure 8:**
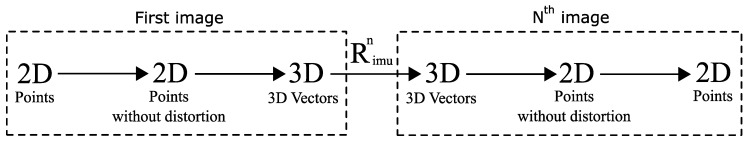
The various steps required to obtain the approximate initial homologous point in another image. $R_{imu}^{n}$ is the 3D rotation obtained by IMU measurements.

Using the internal orientation parameters produced by the optical calibration process and the 3D rotations between poses from the IMU, we can compute the predicted positions of homologous points of detected features in other images as illustrated in Figure 8.

**Figure 9:**
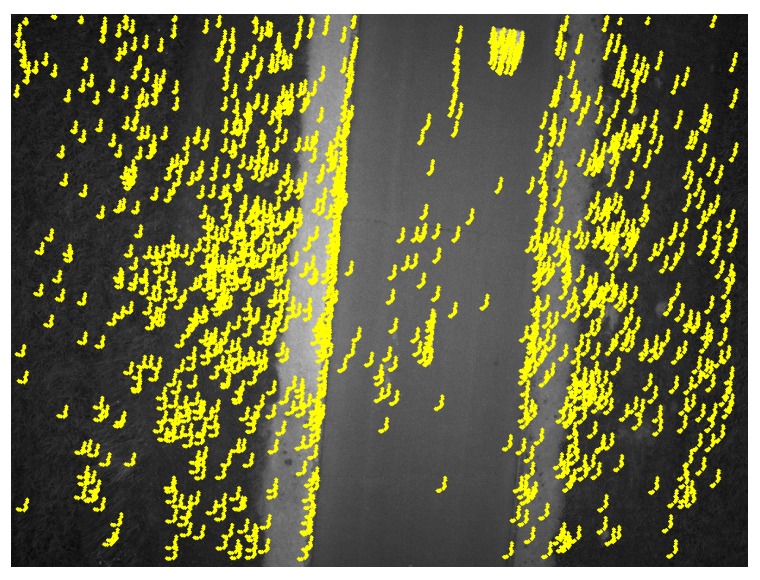
Positions of the features predicted by the IMU for all images.

Let us consider that $P_{i}^{1}\left(u,v\right)$ is a 2D point in the plane of the first image. First, the geometric distortion is removed, then $P_{i}^{1}\left(u,v\right)$ is transformed to a 3D vector $\vec{V_{i}^{1}}\left(x,y,z\right)$ in the camera coordinates system; the origin of this system is at the optical center of the camera. After applying the inverse of the 3D rotation ($R_{imu}^{n}$) to $\vec{V_{i}^{1}}\left(x,y,z\right)$, we obtain a new vector $\vec{V_{i}^{n}}\left(x^{\prime },y^{\prime },z^{\prime }\right)$ that will be projected into the plane of the *N*-th image to obtain the corresponding 2D point $P_{i}^{n}\left(u^{\prime },v^{\prime }\right)$ after applying the geometric distortion. The accuracy of the estimated 3D camera rotation ($R_{imu}^{n}$) depends on the accuracy of the IMU. In fact, the main limitation of low-cost MEMS IMUs is the drift due to accumulated uncorrected bias. Subsequently, this rotation ($R_{imu}^{n}$) only provides an approximate position for homologous points in other images as shown in Figure 9.

**Figure 10:**
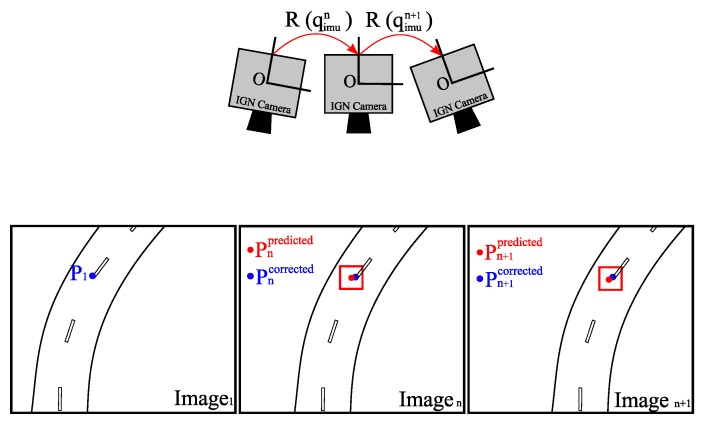
Rotation between three images.

We consider this predicted position as the center of a search area in which the correct position should be found by template matching as illustrated in Figure 10. This step brings the algorithm closer to real-time performance.

### 3.5. Template Matching with ZNCC

**Figure 11:**
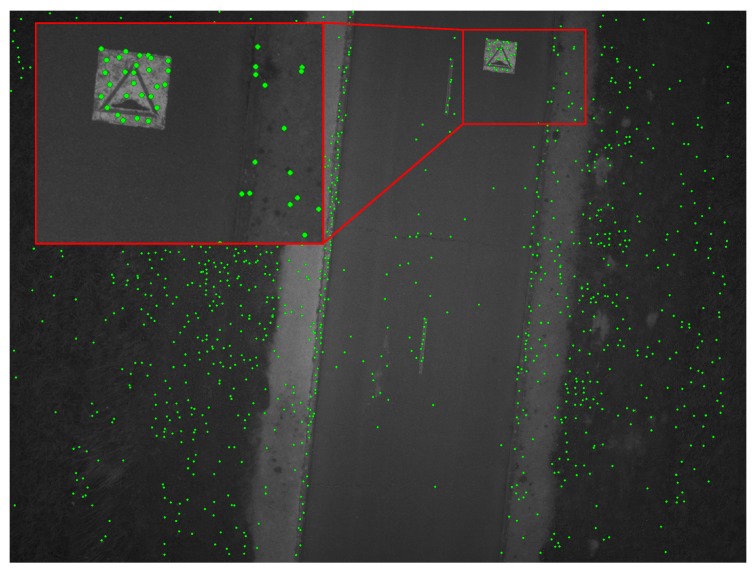
Feature points detected using the FAST algorithm in the first image; the threshold chosen here is 7. The number of grid blocks is defined as 100 × 100; image resolution: 2560 × 1920 pixels.

**Figure 12:**
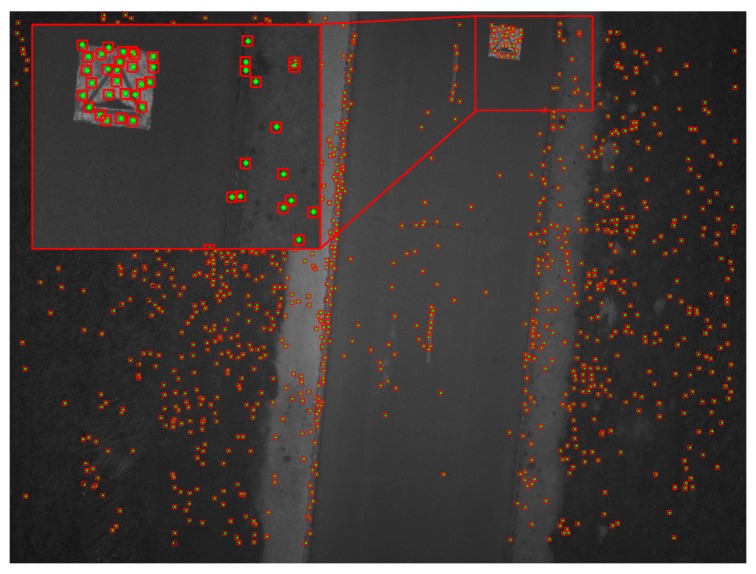
Search areas obtained by the IMU gyrometers between the first image and the 10th image. The green points represent the homologous points obtained by template matching. The size of each search area is 11 × 11 pixels; image resolution: 2560 × 1920 pixels.

Once the centers of search areas in other images have been estimated, the candidate homologous points can be found precisely by template matching as shown in Figure 11 and Figure 12. Due to the fact that the deformations between images are small, the correlation scores are high (close to the maximum correlation value (=1), so we perform here a simple filtering to keep only the pairs with a correlation score higher than 0.85 in order to eliminate possible outliers. We chose ZNCC (Zero mean Normalized Cross-Correlation) [34] because it is a simple and reliable method that can be used to measure the similarity between image patches and is not influenced by variation in brightness and contrast [35]. Whereas ZNCC does not perform well with significant rotation and change of scale between two images, one of our assumptions for this work is that rotation between images is small.

The feature point window and 7 × 7 FAST detector window should have the same size so as to obtain an accurate measurement of the degree of similarity between image patches through the correlation process. The size of search areas depends on the accuracy/bias of IMU gyrometers, which means that the size of search areas should be chosen to be large enough (11 × 11 pixels) between the first and second images to accommodate prediction errors. This size can then be reduced for all other images after eliminating bias. This correlation could be implemented in hardware to enable real-time processing [36].

#### The Sub-Pixel Correlation

One of drawbacks of ZNCC is that its resolution is limited to one pixel, i.e., the peak location coordinates have integer values. Matching precision can, however, be improved directly by interpolating the cross-correlation surface to a higher spatial resolution in order to locate the correlation peak with sub-pixel accuracy [37]. The sub-pixel location can be approximated by fitting a two-dimensional model through the correlation coefficients around the peak location. Several models are proposed in the research community such as parabola fitting and Gaussian fitting [38,39,40]. In our work, we choose the simple parabolic curve fitting model that computes the sub-pixel peak location separately for the two orthogonal directions where the curve reaches its maximum. Results presented in Figure 15 demonstrate that with this simple model, the sub-pixel approach improves the precision and accuracy of ZNCC-based image matching, eliminating an average of 0.15 residual pixels^2^ with respect to the precision of the pixel-level matching, so it is not then necessary to use another complex fitting model. The rounding of uniformly-distributed random variables caused error in the estimation of the variance of 1/12 pixels^2^. Since we have 2 independent variables x and y, the overall error will see its variance increased by 1/6 pixels^2^, that is 0.17. We see that our method corrects nearly all of this noise.

### 3.6. Estimation of Transformation between Images

Now that we have good homologous pairs, the next step is to establish the best estimation of a geometric relation that maps pixel coordinates from one image to another. Two geometric transformations can be established here, 3D rotation between poses and 2D homography between images. Which of these two transformations is selected depends on the real movement of the camera.

3D rotation is used when the UAV is quasi-stationary and only attitude variations are considered, and it is beneficial for improving the robustness of the system because it only has three degrees of freedom. This approach is not optimal if the real movement of the camera is not a pure rotation. Moreover, in this case, it is not possible to recalibrate the IMU gyrometers directly from this transformation.

2D homography is used when the camera movement consists of both rotation and translation. This transformation has eight degrees of freedom, which means that it can partially absorb the translation movement of the camera. On the other hand, this approach is more sensitive to errors and does not work perfectly when the scene is not planar.

#### 3.6.1. Estimation of 3D Rotation Camera Using the Least Squares Method

Once given a set of 3D vectors $\vec{V_{i}^{1}}\left(x,y,z\right)$ and corresponding set of 3D vectors $\vec{V_{i}^{n}}\left(x^{\prime },y^{\prime },z^{\prime }\right)$, the 3D rotation $R_{img}^{n}$ is estimated using the least squares method, which minimizes the errors between estimated 3D vectors $\hat{\vec{V_{i}^{n}}}$ and renormalized 3D vectors $\vec{V_{i,corr}^{n}}$ resulting from the correlation. The IMU can be used to obtain an initial solution, and then, only one iteration is needed to be close enough to the true solution; several more iterations are then used to automatically eliminate outliers due to false correlations.

The relative 3D rotation between $R_{img}^{n}$ and $R_{imu}^{n}$ is used to estimate, in real time, most of the drift of the IMU bias and then improve the accuracy of the estimation of the consecutive 3D rotation $R_{img}^{n+1}$.

#### 3.6.2. Estimation of 2D Homography Using the Least Squares Method

With the 2D planar homography model, geometrical transformation can be calculated easily using the least squares method, with the 2D points obtained by the FAST detector in the first image and their homologous points found by template matching in the *N*-th image. Outliers with errors greater than three pixels are automatically eliminated in order to obtain a robust estimation of the best fitting homography.

### 3.7. Resampling Images and Generating the Stacked Image

We used bilinear interpolation to find the resampled value of each pixel from the first image in the *N*-th images. The stacked intensity values are then saved with a coefficient that can be used to encode the values of the stacked image on 8 bits instead of 12 bits and optionally increase the brightness of the image. This stage is the most computationally-intensive task in the algorithm, as shown in Table 2. However, the process of mapping each pixel from the first image to the other images can be largely accelerated by using a bilinear approximation. This approach consists of dividing the first image into M × N blocks and computing for each block the bilinear function that matches the real transformation on the corners of the block, then using the nearest neighbor resampling method to resample each pixel in other images. Thus, we significantly reduce the number of arithmetic operations and memory access use. This method is very effective in software, and we currently aim to implement it in hardware because it uses less resources (especially DSP resources) and requires less memory access.

## 4. Results

We performed a few experiments to quantify the accuracy and performance of our algorithm. We used the hardware described in Section 3. Several datasets were acquired by the IGN photogrammetric camera, some of which are shown here. Each dataset consists of a sequence of ten images acquired at the maximum frame rate of the CMOS (30 images/s) by transmitting a wireless signal to trigger image acquisition when the UAV is hovering. The value of exposure time of acquisition is set to 1 ms to be closer to hyperspectral images with a low dynamic range.

### 4.1. Timing Results (in Seconds)

All tests were run on two platforms: firstly, a HPZ210 Workstation with an Intel Xeon 3.30-GHz processor and 8.0 GB of RAM; secondly, the photogrammetric IGN camera with 666-MHz dual-core ARM Cortex-A9 processors.

### 4.2. Accuracy of IMU

**Figure 13:**
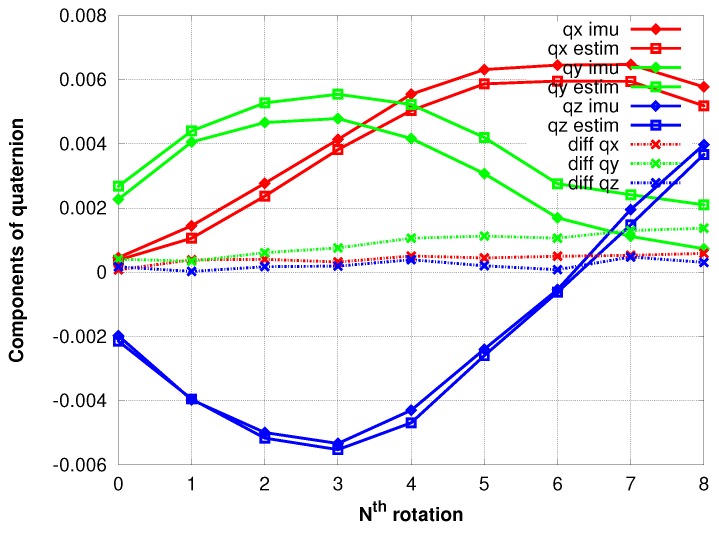
The two 3D rotations of the camera between poses.

Figure 13 shows the 3D rotations respectively experienced by the IMU and estimated by the photogrammetric method. We chose here a dataset in which the bias of IMU drifts is significant.

### 4.3. Accuracy of Transformation Estimation

#### 4.3.1. Case of Pure Rotation Camera Movement

**Figure 14:**
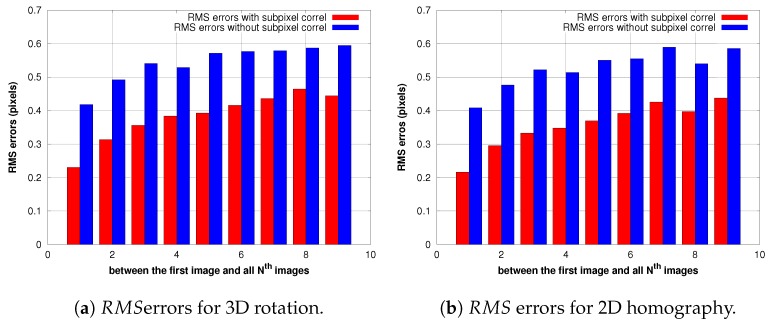
Residuals of the estimation of the 3D rotation and 2D homography.

**Figure 15:**
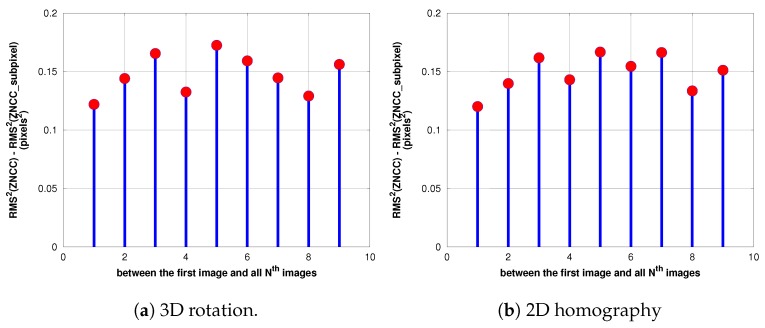
$RMS^{2}$ difference between ZNCC without sub-pixel and ZNCC with sub-pixel accuracy.

**Figure 16:**
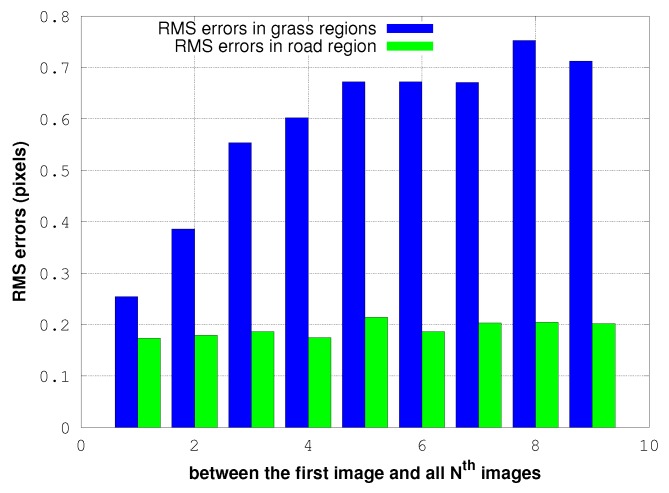
Residuals of the 3D rotation estimation between the first image and all other images in grass and road areas.

Figure 14, Figure 15 and Figure 16 illustrate a case in which the UAV has experienced a pure rotation movement. In order to explain the augmentation of RMS errors over images, we separate the calculation of errors between grass regions and road regions.

#### 4.3.2. Case of Non-pure Rotation Camera Movement

**Figure 17:**
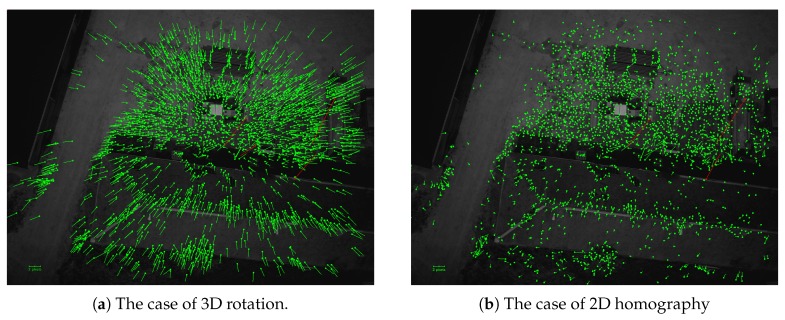
Residues obtained using the transformation estimation between the first image and the 10th image in the case of non-pure rotation mouvement of the camera. Red arrows represent the outliers.

**Figure 18:**
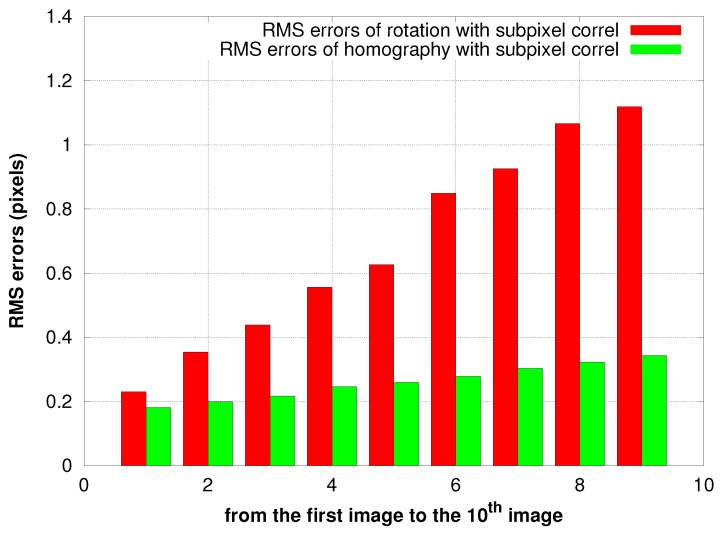
Residuals of the estimation of 3D rotation and 2D homography between the first image and all other images in the case of UAV translation.

Figure 17 and Figure 18 illustrate a case in which the UAV has suffered a Z-translation movement caused by a free-fall.

### 4.4. Quality of Resulting Image

**Figure 19:**
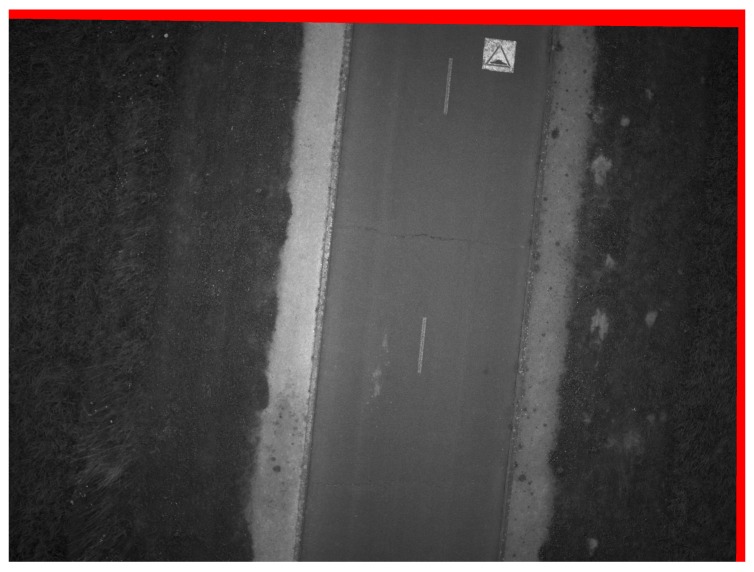
The stacked image. The red part is the non-common part between the first image and the 10th image.

Figure 19 represents the final stacked image resulting from our algorithm. The red part shows the rotation experienced by the camera between the first and last image. It is possible here to use a brightness factor to illuminate the image.

## 5. Discussion

### 5.1. Timing Results

Table 2 shows that the resampling part of the algorithm is the most consuming task for the two types of transformations. Benefiting from the presence of dual-core processors in the camera, we use the OpenMP library to accelerate the image resampling part; the number of threads was set to two; the processing time of this part was reduced by half. The acceleration factor when using the nearest-neighbor resampling method is seven. This part of the process should be accelerated even further with the help of the programmable logic (FPGA) to achieve real-time performance.

### 5.2. Accuracy of IMU

Our results show that the errors of components increase slowly and linearly over time, implying that the bias is constant over the sequence duration. This bias can be then estimated by computing the 3D rotation linking the attitude estimated by the photogrammetric method and the one obtained by the IMU. This rotation can then be used to obtain a better initial rotation for the following images, leading to the reduction of the size of the research areas and then of the correlation time, or to correct the biases’ values in the IMU subsystem; which could be beneficial to other sensors (i.e., LiDAR) on the UAV.

### 5.3. Accuracy of Estimation of Transformations

#### 5.3.1. Case of Pure Rotation Movement of the Camera

For the two transformations, the RMS errors are less than 0.5 pixels for all of the images that are processed. Our results presented in Figure 14 show also a clear improvement of accuracy when we use the sub-pixel correlation for both transformations. Figure 15 demonstrates that the difference in variance between sub-pixel correlation and non-sub-pixel correlation almost equals (2/12), corresponding to the variance in the uniform distribution; which means that the sub-pixel correlation works as expected. Additionally, the estimation of the homography has less residuals than the rotation, consistent with the fact that former has more degrees of freedom.

In both cases, the RMS values of errors increase linearly over images. Figure 16 shows that this is due to the movement of the grass next to the road: the residuals in the road region are constant and significantly lower. This indicates also that the noise of our process is only of the order of 0.2-pixel RMS.

#### 5.3.2. Case of Non-pure Rotation Movement of the Camera

In this case, the estimation of the rotation with our photogrammetric method does not work optimally because the optical center is moving; thus, the residual errors are bigger than expected. They converge to the center of the image as shown in Figure 17a because the UAV is experiencing a free-fall. These residuals, which are not compensated by the rotation, can be absorbed by the 2D homography, which includes a scale factor: Figure 18 demonstrates that the residual errors of the estimated homography are constant over images and much smaller than those of the 3D rotation. However, as expected, they are more important in zones where the surface gets away from the average plan in the scene such as the rooftop shown in Figure 17b. This means that for these parts of the image, the stacked image will be slightly blurred.

Thanks to the RMS error, we have a control quality factor that helps our processing to determine which type of transformation should be used to obtain the best results.

### 5.4. Quality of Resulting Image

**Figure 20:**
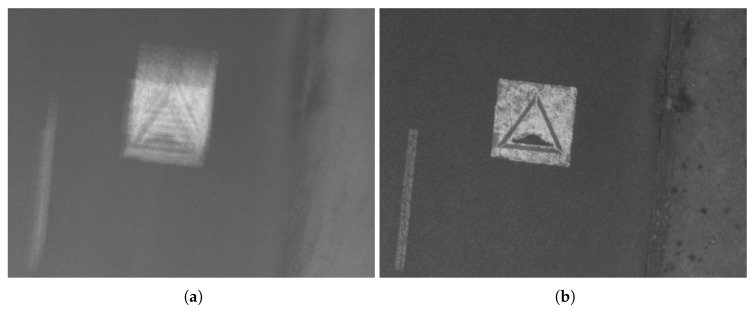
Example illustrating two subsections of two images. (**a**) is obtained by the average of the ten images; (**b**) is the stacked image. The angular motion between images is 1.2 degrees corresponding to 59 pixels.

Figure 19 demonstrates visually that no motion blur mars the stacked image. Figure 20 shows that our method can be used to eliminate the motion blur caused by erratic movement of the camera. Images can be improved to a point where the blur is almost undetectable even if the movement of the camera is complex (Figure 9), where the trajectories of predicted features are not the same over the entire image. Our method can be applied for images that exceed an angular motion blur of much more than several pixels unlike de-convolution methods [14] that have strict limits on the amount of angular motion.

## 6. Conclusions and Future Works

An image processing stacking algorithm has been presented, which consists of several steps. First, feature points are detected in the first image and matched using ZNCC correlation with the help of IMU measurements; then, 3D rotation (and/or 2D homography) between sequential images is estimated using a photogrammetric method, which leads to a better accuracy. This geometrical transformation (3D rotation or 2D homography) is then used to resample images, and 3D rotation in particular can be used to recalibrate the IMU gyrometers by estimating their bias. This algorithm benefits from the presence of an FPGA to speed up some tasks, like detecting feature points. The proposed method was tested with the IGN camera embedded on an UAV, and the experimental results show the efficiency and accuracy of this method when the UAV is quasi-stationary. The IMU data processing part is already real time and can be used for other applications.

In the future, we will implement a real-time version by acceleration of the image resampling part in the FPGA, since this is the most demanding part of processing. We will refine all parameters to obtain good results in less time. We will also integrate the translation of the UAV in our model using GNSS/IMU data, which may later be used to correct translation deformation. Other sensors like LiDAR or other synchronized cameras can benefit from the resulting better quality of the IMU data. In fact, this methodology is intended to be used on an airborne prototype imaging system with different narrow spectral channels composed of 10 synchronized IGN cameras, which will make it possible to obtain images with an equivalent long exposure time. 

## Figures and Tables

**Table 1:** Resources usage of the FAST detector design in Xilinx/Zynq-7030 FPGA.

Resource	Used	Available	Utilization (%)
Slice LUTs	232	78,600	0.30
Slice of 4 registers	472	157,200	0.30
Memory (blocks of 36 KB)	6	265	2.26
Clocking	1	32	3.13

**Table 2:** (1) Initial geometrical transformation with bilinear interpolation; (2) accelerated geometrical transformation using the bilinear function method with bilinear interpolation; (3) accelerated geometrical transformation using the bilinear function method with nearest-neighbor resampling.

	Feature Detection (402 Points)	Matching (402 Points) & Estimation of 3D Rotation	Resampling			
			Rotation			Homography
			(1)	(2)	(3)	(1)
PC (Soft)	0.21	0.08	4.72	0.94	0.66	4.00
PC (Soft/2 Threads)			2.46	0.42	0.38	2.06
Camera (Soft)	1.64	0.83	36.94	7.24	5.28	25.17
Camera (Hard)	0.02					
Camera (Soft/2 Threads)			18.48	3.81	2.65	12.57
